# Factors Associated With Lower Respiratory Tract Infection Among Chinese Students Aged 6–14 Years

**DOI:** 10.3389/fped.2022.911591

**Published:** 2022-06-16

**Authors:** Mei Xue, Qiong Wang, Yicheng Zhang, Bo Pang, Min Yang, Xiangling Deng, Zhixin Zhang, Wenquan Niu

**Affiliations:** ^1^Graduate School, Beijing University of Chinese Medicine, Beijing, China; ^2^Department of Pediatrics, China-Japan Friendship Hospital, Beijing, China; ^3^International Medical Services, China-Japan Friendship Hospital, Beijing, China; ^4^Institute of Clinical Medical Sciences, China-Japan Friendship Hospital, Beijing, China

**Keywords:** lower respiratory tract infection, machine learning, deep learning, factor, performance

## Abstract

**Aims:**

We employed machine-learning methods to explore data from a large survey on students, with the goal of identifying and validating a thrifty panel of important factors associated with lower respiratory tract infection (LRTI).

**Methods:**

Cross-sectional cluster sampling was performed for a survey of students aged 6–14 years who attended primary or junior high school in Beijing within January, 2022. Data were collected *via* electronic questionnaires. Statistical analyses were completed using the PyCharm (Edition 2018.1 x64) and Python (Version 3.7.6).

**Results:**

Data from 11,308 students (5,527 girls and 5,781 boys) were analyzed, and 909 of them had LRTI with the prevalence of 8.01%. After a comprehensive evaluation, the Gaussian naive Bayes (gNB) algorithm outperformed the other machine-learning algorithms. The gNB algorithm had accuracy of 0.856, precision of 0.140, recall of 0.165, F1 score of 0.151, and area under the receiver operating characteristic curve (AUROC) of 0.652. Using the optimal gNB algorithm, top five important factors, including age, rhinitis, sitting time, dental caries, and food or drug allergy, had decent prediction performance. In addition, the top five factors had prediction performance comparable to all factors modeled. For example, under the sequential deep-learning model, the accuracy and loss were separately gauged at 92.26 and 25.62% when incorporating the top five factors, and 92.22 and 25.52% when incorporating all factors.

**Conclusions:**

Our findings showed the top five important factors modeled by gNB algorithm can sufficiently represent all involved factors in predicting LRTI risk among Chinese students aged 6–14 years.

## Introduction

Lower respiratory tract infection (LRTI) is a common infectious disease in pediatric clinics, and it ranks as a leading cause of pediatric deaths (1–3). LRTI places a heavy burden on individuals and public health systems. Pneumonia is the most common form of LRTI, and it, on average around the world, takes the lives of three children every 2 min (4). Global statistics have shown that hospitalization due to pneumonia increased by 2.9 times from 2000 to 2015 (5), and each year 0.65 million children die of LRTI (6, 7). Given the complex pathogenesis of LRTI, there is increasing interest in understanding the causes of LRTI, proposing effective prediction algorithms, and risk-stratifying children who might benefit from close monitoring and timely interventions.

To date, numerous studies have been conducted to identify factors that can better predict LRTI occurrence. Notably, Shi et al. have written an excellent systematic review and meta-analysis focusing on factors susceptible to respiratory syncytial virus associated acute LRTI among young children, and they found that comorbidity, congenital heart disease, prematurity, and younger age were associated with poor outcomes (8). More recently, we, among 7,222 preschool-aged children, have identified five factors of significance that were associated in a synergistic manner with recurrent respiratory tract infection (9). Thus far, no consensus exists on how many LRTI-susceptibility factors are actually involved and how they act. The reasons for this challenge are partly due to the difficulty in delineating more complicated and nuanced relationship among factors to predict LRTI when adopting traditional statistical methods (such as Logistic regression analysis), which involve only one input-output layer and accommodate relatively small amounts of variation. To overcome this challenge, more advanced machine-learning methods have been developed and successfully applied in a variety of clinical settings (10–12). To our knowledge, there is to date no application of machine-learning methods in the field of LRTI.

To fill this gap in knowledge and generate more information for future studies, we attempted to employ machine-learning methods to manage data from a large survey on students 6–14 years of age, with the goal of identifying and validating a thrifty panel of important factors associated with LRTI, and meanwhile selecting the optimal algorithm for possible clinical application.

## Methods

### Study Design

This survey was performed following a cross-sectional cluster sampling design within January, 2022 in Pinggu district, Beijing. The Ethics Committee of Beijing University of Chinese Medicine reviewed and approved the protocols of this survey, which was implemented according to the Declaration of Helsinki.

### Study Participants

Study participants are consisted of students aged 6–14 years and attending primary school or junior high school at the time of survey. The parents or guardians of participating students provided electronic signature consenting to the participation of this survey, and importantly there are opt-out clauses in our consent form.

A total of 26 schools in Pinggu district were randomly selected, including eight primary schools and 18 junior high schools. The total number of registered students in these 26 schools was 11,633. The self-designed questionnaire was generated by the “Wenjuanxing” website (https://www.wenjuan.com/), an online platform in mainland China in the form of QR code. The QR-coded questionnaire can be easily recognized by common smart phones on the market, and it was sent to the parents or guardians of 11,633 students by their teachers-in-charge *via* the “WeChat” social media APP.

### Data Collection

The questionnaire used in this survey was circulated to the parents or guardians of a small number of students (*N* = 120), and the reliability coefficient (alpha) was over 0.85. Specifically, items in the questionnaire were related to both students themselves and their parents from multiple aspects, and were downloaded into a Microsoft Office Excel™ spreadsheet.

From students, information was collected on age, sex, nationality, waist-hip rate, body mass index (BMI), pregnancy order, delivery order, twin birth, delivery mode, gestational age, birth weight, birth body length, infancy feeding, breastfeeding duration, pure breastfeeding, pure breastfeeding time, time of adding solid-food, stool frequency, and stool consistency, as well as lifestyle-related factors including eating speed, fall asleep time, sleep duration, sitting time, screen time (time of watching TV or playing video games), daily time of outdoor activities, sleeping with the light on, using plastic tableware, using make-up, as well as the weekly intake frequencies of dietary fiber, out-of-season fruit, animal protein, soy protein, milk, dietary supplement, food containing preservative, fast food, snacks, sweet food, night meals, and picky eating frequency per week. In addition, the episodes of LRTI over the past year, chronic diseases, dental caries, and rhinitis allergy (including foods or drugs) were also recorded.

From parents, information on BMI, bearing age and education of both parents, family income (RMB per year), number of relatives with hypertension and diabetes was collected.

### Quality Control

Quality of survey data was strictly controlled. Specifically, school-healthcare physicians and teachers in charge of class were trained to understand the detailed procedure of this survey and each item in the questionnaire. They were responsible for assisting the parents or guardians of participating students to fill out this questionnaire. As the survey ended, data were downloaded from the “Wenjuanxing” platform, and each item was rigorously checked. In the case of missing values and obvious outliers, school-healthcare physicians and teachers in charge of class were requested to contact the parents or guardians of participant students to provide or confirm relevant information.

### LRTI Definition

Clinically, LRTI refers to the infection of the lung tissue or tracheobronchitis below the throat, and it is usually caused by viruses or bacterial microorganisms from the mouth and upper respiratory tract spreading down the respiratory tract (13). In this survey, LRTI refers to the occurrence of LRTI diagnosed by doctors in the past year, whose hospital or outpatient clinic diagnosis cases and information were confirmed by teachers in charge of class. If there was any disagreement, our team would further verify carefully regarding the content of the inquiry included symptoms and related diagnosis and treatment.

### Definitions of Other Items

Allergic rhinitis was diagnosed based on previous medical records, and food/drug allergy was identified by questions related to physician diagnosis in accordance with the International Study of Asthma and Allergies in Childhood (ISAAC) questionnaire (14). Dental caries was recorded, and medical history of children referred to chronic kidney diseases, congenital heart disease, hypothyroidism, and other chronic diseases.

BMI was calculated as body weight divided by height squared (kg/m^2^). Body weight and height were measured by school-healthcare physicians. Infancy feeding included pure breastfeeding, partial breastfeeding, and non-breastfeeding. Gestational age, breastfeeding duration and solid food consumption age were recorded in months. Delivery mode included vaginal delivery and cesarean section. Stool frequency was classified into 1–2 times per day, 3–4 times per day, more than 4 times per day, 2–3 times per week and 0 or once per week. Stool consistency was classified into four categories according to the Bristol Stool Form Scale (BSFS). Lifestyle-related factors included sleep habits, daily activity habits, sitting habits, and eating habits. Specifically, sleep duration, sitting time, screen time, daily duration of outdoor activities, and recorded in hours were, respectively, calculated as the sum of both on workdays ×5 and weekends ×2 divided by 7. The weekly intake frequency of eating the following foods (dietary fiber, out-of-season fruit, animal protein, soy protein, milk, dietary supplement, food containing preservative, fast food, snacks, and sweet food) was classified as every day, three or more times per week, once or twice per week and hardly. The frequency of the following behaviors (sleeping with the light on, using plastic tableware, using make-up night meals, and picky for foods) was categorized into four groups, that is, every day, three or more times per week, once or twice per week, and hardly.

For parents or guardians, maternal and paternal BMI was calculated from self-reported body weight and height. Education was categorized as middle school degree or below, high school degree, and college degree or above. Family income (RMB per year) was categorized as <100,000, 100,000–300,000, and ≥300,000. The relative diseases referred to as diabetes mellitus or hypertension diagnosed by doctors from tertiary hospitals.

### Statistical Analyses

If the missing percent of each item in the questionnaire exceeds 30%, this item was removed from the final analysis. The expression of continuous factors is mean (standard deviation) if no deviation from normal distribution is observed, and median (interquartile range) otherwise. The expression of categorical factors is count (percent). Two-group (students with and without LRTI within the last year) comparison was done using *t*-test for normally distributed factors, rank-sum test for skewed factors, and χ^2^-test for categorical factors.

To ensure the reproducibility of machine-learning models, data from 11,308 students were randomly divided into the training set (60%, *N* = 6,785 students) and the testing set (40%, *N* = 4,523 students). The training group is used to construct the machine-learning algorithms, and the testing group is used to test the reproducibility of these algorithms. In this study, 11 machine-learning algorithms were trained, including Logistic regression, random forest, support vector machine (SVM), decision tree, K-nearest neighbors (KNN), gradient boosting machine (GBM), light gradient boosting machine (LGBM), extreme gradient boosting machine (XGBoost), Gaussian naive Bayes (gNB), multinomial naive Bayes (mNB), and Bernoulli naive Bayes (bNB). Meanwhile, both hard and soft voting classifications were calculated based on the 11 machine-learning algorithms. The performance of each algorithm was evaluated from five aspects, that is, accuracy, precision, recall, F1 score and AUROC. By definition, accuracy refers to the rate of correct prediction, and precision measures the ability to target actual positive observations. Recall reflects the capability to predict actual positivity correctly. F1 score, calculated as the harmonic mean between precision and recall, takes both false positives and false negatives into account. AUROC is proposed as a summarized accuracy index, with a higher value indicating a higher probability of having the characteristic under study. The optimal algorithm was selected after comprehensive evaluation of above five aspects.

To narrow the range of contributing factors, the importance of each factor was calculated using the SHAP (SHapley Additive exPlanation) tool. After ordering the importance of all variables from the highest to the lowest, the prediction performance of an increasing number of top factors was appraised by accuracy, precision, and AUROC, upon which the minimal number of important variables was determined. Further, the contribution of these variables was compared with that of all variables in terms of model accuracy and model loss under study by using the deep-learning sequential model with three types of optimizers (adaptive moment estimation, root mean square prop, and stochastic gradient descent).

The statistical handling was done by using the community PyCharm (Edition 2018.1 x64) on the Windows 10 system with the Python (Python Software Foundation) software (Version 3.7.6). Missing data were supplemented according to the multiple imputation procedure, which was implemented by the MICE package in the R programming environment (Version 4.1.1).

## Results

### Baseline Characteristics

After excluding invalid questionnaires, data from 11,308 students (5,527 girls and 5,781 boys) were analyzed finally, with response rate of being 98%. There were 909 students who had experienced LRTI during the last year, and so the prevalence of LRTI in this student population was 8.01%.

The baseline characteristics of all participating students are presented in Table 1 according to the presence and absence of LRTI.

**Table 1:** The baseline characteristics of participating students according to lower respiratory tract infection.

**Factors under study**	**Absence of LRTI**	**Presence of LRTI**	***P***
	**(*n* = 10,399)**	**(*n* = 909)**	
**Baseline factors**			
Sex (%)			0.080
Boys	5,291 (50.9)	490 (53.9)	
Girls	5,108 (49.1)	419 (46.1)	
Age (months)	128 (105,153)	110 (94,132)	<0.001
Waist-hip rate	0.85 (0.79, 0.91)	0.86 (0.81, 0.92)	<0.001
BMI	18.83 (16.14, 22.52)	18.56 (15.86, 22.35)	0.045
Gestational age	39 (38, 40)	39 (38, 40)	0.119
Twins (%)			0.554
No	10,137 (97.5)	889 (97.8)	
Yes	262 (2.5)	20 (2.2)	
Chronic disease (%)			0.839
No	10,259 (99.5)	880 (99.4)	
Yes	53 (0.5)	5 (0.6)	
Number of dental caries (%)			<0.001
0	5,536 (53.2)	383 (42.1)	
1	1,412 (13.6)	130 (14.3)	
2	1,650 (15.9)	165 (18.2)	
3	730 (7.0)	90 (9.9)	
4	536 (5.2)	67 (7.4)	
≥5	535 (5.1)	74 (8.1)	
Rhinitis (%)			<0.001
No	7,979 (76.7)	461 (50.7)	
Yes	2,420 (23.3)	448 (49.3)	
Eczema (%)			<0.001
No	8,313 (79.9)	577 (63.5)	
Yes	2,086 (20.1)	332 (36.5)	
Allergy (food/drug) (%)			<0.001
No	9,224 (88.7)	721 (79.3)	
Yes	1,175 (11.3)	188 (20.7)	
**Lifestyle-related factors**			
Eating speed (minutes)	16.67 (13.33, 20.00)	16.67 (13.33, 21.67)	0.035
Fall asleep time (hours per day)	10.00 (9.00, 10.00)	10.00 (9.00, 10.00)	0.002
Sleep duration (hours per day)	9.00 (8.29, 9.29)	9.00 (8.29, 9.29)	0.002
Sitting duration (hours per day)	5.71 (3.43, 7.43)	5.43 (2.79, 7.00)	<0.001
Screen time (hours per day)	1.29 (0.64, 1.86)	1.29 (0.79, 1.57)	0.708
Daily time of outdoor activities (hours per day)	1.29 (1.00, 1.64)	1.29 (1.00, 1.57)	0.952
Weekly intake frequency of dietary fiber (%)			0.665
Every day	235 (2.3)	16 (1.8)	
≥3 times per week	1,656 (15.9)	154 (16.9)	
1–2 times per week	2,976 (28.6)	262 (28.8)	
Hardly	5,532 (53.2)	477 (52.5)	
Weekly intake frequency of out-of-season fruit (%)			0.281
Every day	1,427 (13.7)	104 (11.4)	
≥3 times per week	3,697 (35.6)	327 (36.0)	
1–2 times per week	2,877 (27.7)	261 (28.7)	
Hardly	2,398 (23.1)	217 (23.9)	
Weekly intake frequency of animal protein (%)			0.465
Every day	154 (1.5)	8 (0.9)	
≥3 times per week	1,475 (14.2)	133 (14.6)	
1–2 times per week	3,311 (31.8)	298 (32.8)	
Hardly	5,459 (52.5)	470 (51.7)	
Weekly intake frequency of soy protein (%)			0.115
Every day	820 (7.9)	77 (8.5)	
≥3 times per week	4,212 (40.5)	393 (43.2)	
1–2 times per week	2,851 (27.4)	250 (27.5)	
Hardly	2,516 (24.2)	189 (20.8)	
Weekly intake frequency of milk (%)			0.033
Every day	305 (2.9)	36 (4.0)	
≥3 times per week	1,322 (12.7)	104 (11.4)	
1–2 times per week	2,526 (24.3)	250 (27.5)	
Hardly	6,246 (60.1)	519 (57.1)	
Weekly intake frequency of dietary supplement (%)			0.120
Every day	8,592 (82.6)	769 (84.6)	
≥3 times per week	990 (9.5)	78 (8.6)	
1–2 times per week	355 (3.4)	35 (3.9)	
Hardly	462 (4.4)	27 (3.0)	
Weekly intake frequency of food containing preservative (%)			0.006
Every day	5,770 (55.5)	460 (50.6)	
≥3 times per week	3,517 (33.8)	335 (36.9)	
1–2 times per week	690 (6.6)	81 (8.9)	
Hardly	422 (4.1)	33 (3.6)	
Weekly intake frequency of fast food (%)			0.021
Every day	4,716 (45.4)	367 (40.4)	
≥3 times per week	4,919 (47.3)	464 (51.0)	
1–2 times per week	480 (4.6)	53 (5.8)	
Hardly	284 (2.7)	25 (2.8)	
Weekly intake frequency of snacks (%)			0.071
Every day	2,106 (20.3)	162 (17.8)	
≥3 times per week	5,762 (55.4)	502 (55.2)	
1–2 times per week	1,769 (17.0)	182 (20.0)	
Hardly	762 (7.3)	63 (6.9)	
Weekly intake frequency of sweet food (%)			0.048
Every day	2,091 (20.1)	148 (16.3)	
≥3 times per week	5,947 (57.2)	539 (59.3)	
1–2 times per week	1,774 (17.1)	166 (18.3)	
Hardly	587 (5.6)	56 (6.2)	
Weekly intake frequency of night meals (%)			0.027
Every day	5,507 (53.0)	440 (48.4)	
≥3 times per week	2,942 (28.3)	287 (31.6)	
1–2 times per week	1,068 (10.3)	90 (9.9)	
Hardly	882 (8.5)	92 (10.1)	
Daily time of sleeping with the light on (%)			0.259
Every day	9,004 (86.6)	775 (85.3)	
≥3 times per week	586 (5.6)	56 (6.2)	
1–2 times per week	260 (2.5)	32 (3.5)	
Hardly	549 (5.3)	46 (5.1)	
Picky eating frequency per week (%)			0.001
Every day	5,293 (50.9)	407 (44.8)	
≥3 times per week	2,993 (28.8)	281 (30.9)	
1–2 times per week	1,141 (11.0)	109 (12.0)	
Hardly	972 (9.3)	112 (12.3)	
Daily time of using plastic tableware (%)			0.154
Every day	6,842 (65.8)	566 (62.3)	
≥3 times per week	2,063 (19.8)	197 (21.7)	
1–2 times per week	631 (6.1)	57 (6.3)	
Hardly	863 (8.3)	89 (9.8)	
Daily time of using make-up (%)			0.876
Every day	9,564 (92.0)	836 (92.0)	
≥3 times per week	466 (4.5)	44 (4.8)	
1–2 times per week	141 (1.4)	10 (1.1)	
Hardly	228 (2.2)	19 (2.1)	
Stool frequency (%)			0.284
1–2 times per day	7,700 (74.0)	697 (76.7)	
3–4 times per day	345 (3.3)	23 (2.5)	
≥4 times per day	323 (3.1)	32 (3.5)	
2–3 times per week	1,719 (16.5)	133 (14.6)	
0 or once per week	312 (3.0)	24 (2.6)	
Stool consistency (%)			0.022
Separate hard lumps, like nuts	227 (2.2)	27 (3.0)	
Sausage-shaped but lumpy	1,383 (13.3)	145 (16.0)	
Like a sausage or snake but with cracks on its surface	1,899 (18.3)	175 (19.3)	
Like a sausage or snake, smooth and soft, fluffy pieces, watery	6,890 (66.3)	562 (61.8)	
**Fetal and neonatal factors**			
Pregnancy order (%)			0.779
1	6,773 (65.4)	587 (64.7)	
≥2	3,626(34.6)	322(35.3)	
Delivery order (%)			0.095
1	8,728 (84.2)	782 (86.5)	
≥2	1,671 (15.8)	127 (13.5)	
Delivery mode (%)			0.007
Vaginal delivery	4,996 (48.0)	394 (43.3)	
Cesarean section	5,403 (52.0)	515 (56.7)	
Birth weight (g)	3,369.62 (455.48)	3,351.91 (453.43)	0.283
Birth body length (cm)	50.77 (2.62)	50.92 (2.58)	0.096
Infancy feeding (%)			0.001
Pure breastfeeding	6,056 (58.2)	471 (51.8)	
Partial breastfeeding	3,177 (30.6)	322 (35.4)	
Non-breastfeeding	1,166 (11.2)	116 (12.8)	
Breastfeeding duration	8.00 (0.00, 12.00)	6.00 (0.00, 13.00)	0.055
Time of adding solid-food	6.00 (6.00, 7.00)	6.00 (6.00, 7.00)	0.318
**Family-related factors**			
Paternal BMI	25.62 (23.46, 27.78)	25.83 (23.66, 27.78)	0.333
Maternal BMI	22.86 (20.81, 25.39)	22.86 (20.96, 25.81)	0.134
Bearing age of the father	27.58 (25.67, 30.08)	27.42 (25.58, 29.58)	0.244
Bearing age of the mother	26.58 (24.50, 28.83)	26.62 (24.75, 28.67)	0.538
Paternal age	39.16 (4.28)	37.95 (4.09)	<0.001
Maternal age	37.84 (4.04)	36.80 (3.79)	<0.001
Menarche	13.54 (1.60)	13.52 (1.59)	0.74
Maternal education (%)			<0.001
Middle school degree or below	1,661 (16.0)	103 (11.3)	
High school degree	3,060 (29.4)	232 (25.5)	
College degree or above	5,678 (54.6)	574 (63.1)	
Paternal education (%)			<0.001
Middle school degree or below	1,692 (16.3)	115 (12.7)	
High school degree	3,778 (36.3)	289 (31.8)	
College degree or above	4,929 (47.4)	505 (55.6)	
Family income (RMB per year) (%)			0.010
<100,000	4,888 (47.0)	384 (42.2)	
100,000–300,000	4,641 (44.6)	453 (49.8)	
≥300,000	870 (8.4)	72 (7.9)	
Number of relatives with hypertension			0.002
0	4,849 (46.6)	364(40.0)	
1	2,448 (23.5)	226 (24.9)	
2	1,888 (18.2)	202 (22.2)	
3	851 (8.2)	81 (8.9)	
4	363 (3.5)	36 (4.0)	
Number of relatives with diabetes			<0.001
0	7,086 (68.1)	549 (60.4)	
1	2,447 (23.5)	256 (28.2)	
2	682 (6.6)	84 (9.2)	
3	140 (1.3)	13 (1.4)	
4	44 (0.4)	7 (0.8)	

*Continuous data are expressed as mean (standard deviation) or median (interquartile range). Categorical data are expressed as count (percentage). For continuous data, P for comparison between children with lower respiratory tract infection and non-lower respiratory tract infection was derived by t test for normally distributed data, by rank-sum test for skewed data, and by χ^2^-test for categorical data*.

*BMI, body mass index*.

### Selection of Optimal Machine-Learning Algorithm

**Figure 1:**
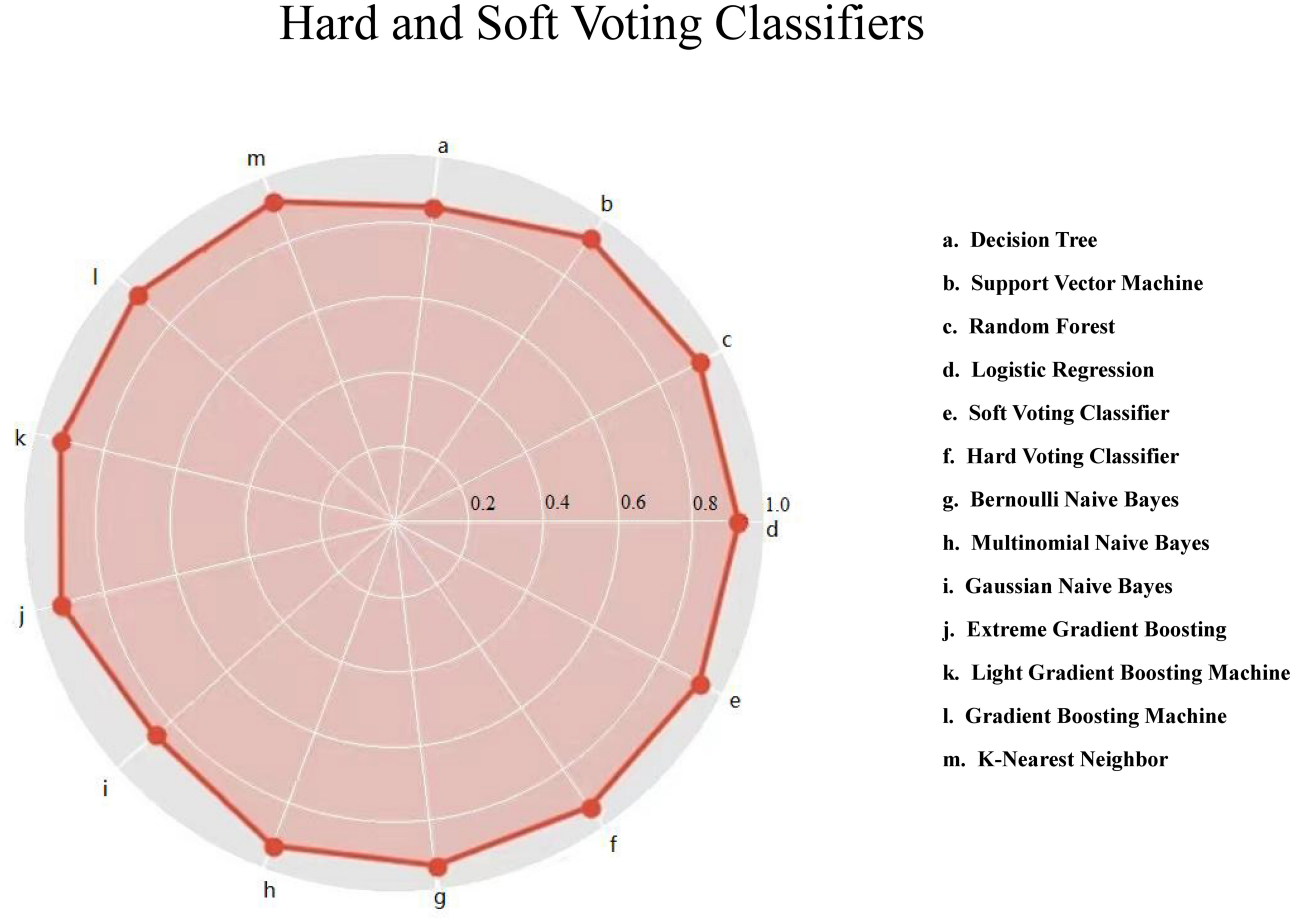
Hard and soft voting classifications based on 11 machine-learning algorithms for lower respiratory tract infection. The red solid circle represents the accuracy.

Figure 1 displays the prediction accuracy of 11 machine-learning algorithms, along with the hard and soft voting classifications. Besides accuracy, 4 other aspects of model performance are provided in Table 2, including precision, recall, F1 score, and AUROC. After taking the five aspects into consideration, gNB algorithm outperformed the other machine-learning algorithms. The gNB algorithm had accuracy of 0.856, precision of 0.140, recall of 0.165, F1 score of 0.151, and AUROC of 0.652.

**Table 2:** Prediction performance of 11 machine learning algorithms for lower respiratory tract infection using accuracy, precision, recall, F1 score and area under the receiver operating characteristic curve (AUROC).

**Algorithms**	**Accuracy**	**Precision**	**Recall**	**F1**	**AUROC**
Logistic regression	0.922	<0.001	<0.001	<0.001	0.710
Decision tree	0.850	0.120	0.148	0.133	0.528
Support vector machine	0.922	<0.001	<0.001	<0.001	0.588
Random forest	0.922	<0.001	<0.001	<0.001	0.654
K-nearest neighbor	0.922	<0.001	<0.001	<0.001	0.514
Gradient boosting machine	0.921	0.143	0.003	0.006	0.682
Extreme gradient boosting	0.918	0.257	0.026	0.047	0.510
Light gradient boosting machine	0.920	0.083	0.003	0.006	0.643
Gaussian naive Bayes	0.856	0.140	0.165	0.151	0.652
Multinomial naive Bayes	0.922	1.000	0.003	0.006	0.663
Bernoulli naive Bayes	0.922	1.000	0.003	0.006	0.682

*AUROC, area under the receiver operating characteristic curve*.

### Importance Ranking and Appraisal

**Figure 2:**
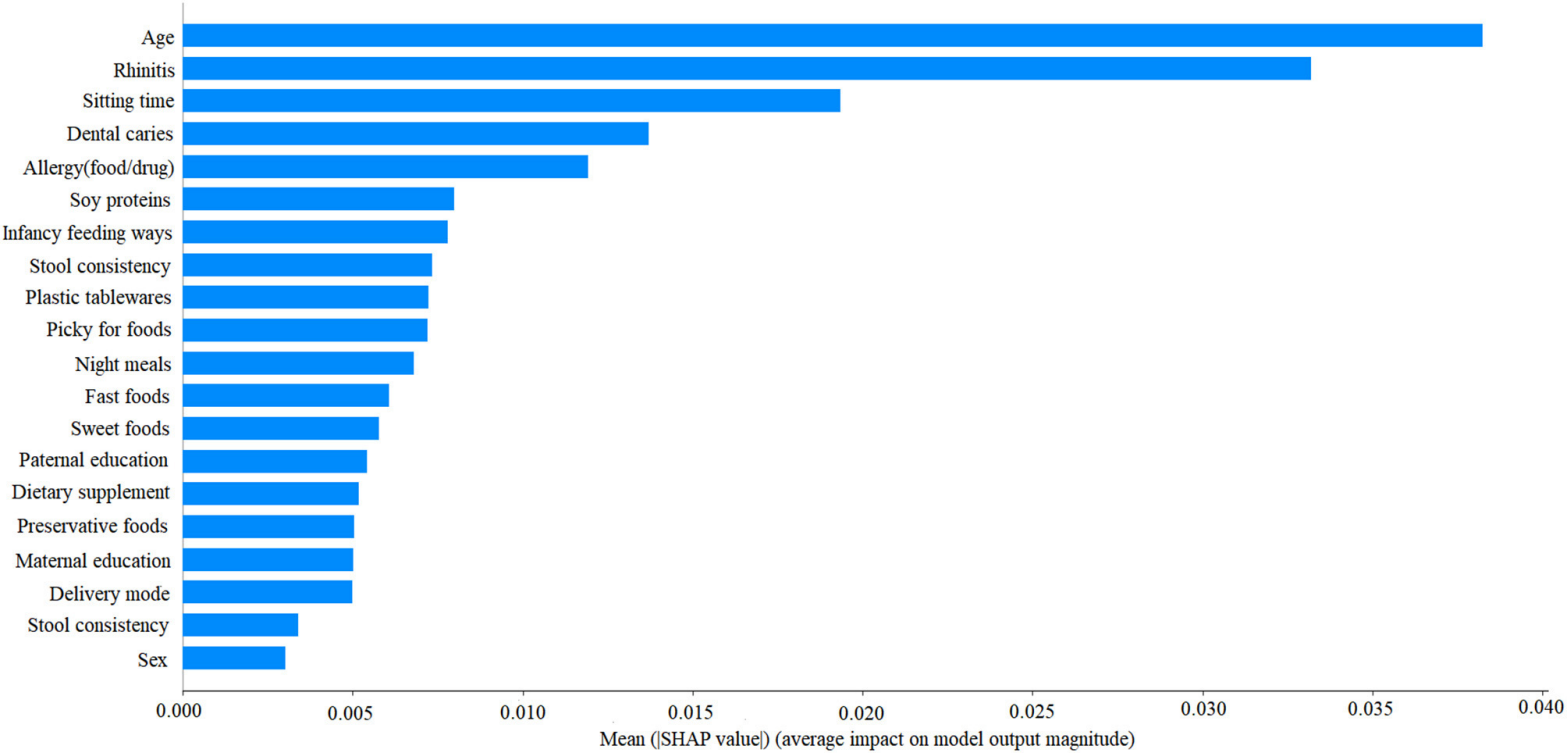
The ranking importance of top 20 factors for lower respiratory tract infection.

To evaluate the contribution of all factors to LRTI prediction, the importance of each factor was gauged and ranked. The importance of top 20 factors is illustrated in Figure 2.

By using the optimal gNB algorithm, the cumulative performance of top 10 factors according to the descending importance was calculated (Table 3). By comparison, the top five important variables, including age, rhinitis, sitting time, dental caries, and allergy, had decent prediction performance.

**Table 3:** Distributions of areas under the receiver operating curve (AUROC), accuracy and precision with the cumulating number of top 10 important factors in an ascending order.

**Number of top 10 factors in rank**	**AUROC**	**Accuracy**	**Precision**
			
1	0.6527	0.9221	<0.0001
2	0.6714	0.9221	<0.0001
3	0.6795	0.8779	0.1428
4	0.6729	0.8896	0.1474
5	0.6914	0.8890	0.1559
6	0.6883	0.8846	0.1487
7	0.6859	0.8828	0.1523
8	0.6867	0.8830	0.1529
9	0.6858	0.8819	0.1472
10	0.6835	0.8806	0.1518

*AUROC, area under the receiver operating characteristic curve*.

### Confirmation of Top Important Factors

To further ascertain the contribution of these top five factors, the deep-learning sequential model was employed by comparing that of all factors under study (Table 4). By comparison, the top five factors had model performance comparable to all involved factors. For example, using the stochastic gradient descent optimizer, the accuracy and loss was 92.26 and 25.62% when modeling the top five factors, and 92.22 and 25.52% after pooling all factors. Thus, the thrifty panel of top factors was established, and these factors can be used to predict the probability of LRTI under the gNB algorithm.

**Table 4:** Model loss and accuracy for deep-learning sequential model using three optimizers in both training and testing groups.

**Optimization algorithms**	**Training group**		**Testing group**	
	**Loss**	**Accuracy**	**Loss**	**Accuracy**
**All factors**				
Adam	23.04%	91.94%	27.62%	92.26%
RMSprop	24.89%	91.92%	26.68%	92.29%
SGD	26.05%	91.77%	25.62%	92.26%
**Top 5 factors**				
Adam	25.94%	91.96%	25.61%	92.22%
RMSprop	27.60%	91.50%	25.65%	92.22%
SGD	26.52%	91.75%	25.52%	92.22%

*Adam, adaptive moment estimation; RMSprop, root mean square prop; SGD, stochastic gradient descent*.

## Discussion

In this cross-sectional analysis on 11,308 Chinese students aged 6–14 years, we attempted to identify and validate a thrifty panel of important variables after comparing the performance of multiple machine-learning algorithms. Importantly, we have teased out the optimal machine-learning model, gNB algorithm, and identified five top important variables that can predict the occurrence of LRTI with performance parallel to that of all variables under study. Moreover, the contribution of the five top important variables to model prediction was further validated by deep-learning model, indicating the robustness and reliability of our findings. To the best of our knowledge, this is thus far the first report that has explored the risk profiles of LRTI in Chinese students over 5 years of age in the medical literature.

More recently, artificial intelligence techniques represented by machine/deep learning have been extensively applied to a growing number of studies to assist or partly replace clinicians in decision making (10–12). As an extension of our previous work adopting traditional statistical methods (line regression and Logistic regression) when modeling, we in this study employed the more advanced machine-learning methods to tease out the optimal algorithm and deep-learning models to validate the contribution of the thrifty panel of important LRTI-susceptibility factors selected by the machine-learning methods. Notably, we narrowed down the list of potential candidate factors, and found that five of these factors, including age, rhinitis, sitting time, dental caries, and allergy, were sufficient to predict the likelihood of LRTI, with decent performance. The modeling of the five factors using the gNB algorithm can be applied in the practical settings to help parents and school-healthcare physicians to monitor the likelihood of having LRTI for early prevention and timely intervention. Our findings are clinical and biologically plausible. It is reasonable to expect that young age is often linked to less mature function, which makes younger students more susceptible to the development of LRTI and associated symptoms. Moreover, allergy to foods and drugs was also identified as a risk-conferring factor for LRTI, and this issue deserves special attention, as the prevalence of allergy in children is steadily increasing around the global (15). Currently, there is no direct evidence for the association of food allergy with LRTI; however, some studies have shown that a variety of respiratory symptoms triggered by foods occurred in up to half of patients (16, 17). Respiratory manifestations of food allergy, an immunoglobulin E-mediated immune responses, arise from damage to the epithelial surfaces of the lungs on account of the epithelium of the lungs being a sensor of environmental stimuli (18–22). Given the important contribution of food allergy to LRTI prediction in the present study, it is reasonable to speculate that susceptibility to respiratory infection might be due to damage of respiratory epithelium caused by food allergy. As demonstrated by James et al. (23) and Larsen et al. (24), increased adherence of pathogens to inflamed respiratory epithelium, increased mucosal permeability, or altered immune response to certain viral and bacterial pathogens can increase the vulnerability to respiratory infection. In a separate study, Vermeulen and Kuehn found that by contrast with non-allergic peers, one of the allergens in allergic rhinitis was food allergens and young children who were sensitized to foods were more likely to induce allergic rhinitis afterwards (25, 26). As such, it is highly recommended for parents to take their children who are allergic to foods to see a pediatrician or allergy specialist for regular intervention with aging. Deeper insights into the independent or combined pathogenicity between rhinitis and food allergy for LRTI were unclear. Nevertheless, more investigations to fully understand the mechanisms of LRTI pertaining to food allergy with or without rhinitis are challenging.

Further, our study indicated that dental caries was a significant contributor to LRTI, and it is notable that more than half of studies (57.9%) who were once diagnosed as LRTI by clinicians had one or more dental caries. This finding was in agreement with that of Mehtonen et al. (27), who found that dental caries was associated with an increased occurrence of LRTI based on a 20-year follow-up of a prospective cohort including children born in Espoo. It is well known that dental caries appears at the beginning of the respiratory system at the mouth and lower respiratory infections deeper in the respiratory tract, and oral cavity harbors one of the most complex microbiomes in the body. Possible mechanisms behind the association between oral health and pneumonia were described by many researchers (28, 29). For example, Thoden van Velzen et al. (30) defined dental plaque as one of the important causes of dental caries and it served as a persistent reservoir for potential pathogens, both oral and respiratory bacteria. Another two studies also reported that oral bacteria in the dental plaque would shed into the saliva and were aspirated into the lower respiratory tract to influence the initiation or progression of LRTI conditions such as pneumonia (31, 32). Hence, for practical reasons, there is necessity to highlight the importance of keeping dental health and reducing LRTI risk.

It is also worth noting that sitting time was found to be associated with the occurrence of LRTI in this study. Sedentary behaviors are predominate in modern life, but adverse effects of these behaviors haven't been completely understood in students. Prolonged sitting time could cause reduced physical activities, which can affect multiple aspects of immune response (33). Evidence from a prospective US cohort indicated that prolonged sitting time increased the chance of pneumonitis due to solids and liquids (34). Other studies showed that regular physical activity was conducive to decreasing mortality and morbidity for influenza and pneumonia (35–37), strengthening the findings of this study. To this point, it is encouraging to elongate physical exercise and outdoor activities of students by reducing sitting time, which can, at least in part, prevent the development of LRTI.

### Strengths and Limitations

Strengths of this study include a large-scale student population from 26 schools in Beijing, a high questionnaire response rate, a wide coverage of potentially candidate factors associated with LRTI, and a comprehensive analysis of contributing predictors for LRTI in students aged 6–14 using advanced artificial intelligence techniques.

Some limitations should be acknowledged when interpreting our findings. Firstly, due to the cross-sectional design of this survey, causality cannot be established. Secondly, our study was based on data from students 6–14 years of age living in a district of Beijing, and extrapolation of our findings to other regions or races should be made with caution. Thirdly, in this survey, data were collected *via* parents-reported electronic questionnaires, which might yield risk for recall or reporting bias, although strict quality control was implemented. Additionally, items analyzed are more general, and some transient factors such as quick weather change from warm to cold and severe air pollution that were found to be susceptible to respiratory infection (38, 39) are not collected in this survey. We agree that further incorporation of more factors is necessary to improve model precision and recall, which are relatively low, even under the optimal gNB algorithm. Our findings presented here are preliminary, and future work will entail refining our model by incorporating more data in other independent groups.

## Conclusions

Our findings showed that gNB algorithm outperformed other machine-learning algorithms, and the top five important factors can sufficiently represent all involved factors in predicting the risk of LRTI in Chinese students aged 6–14 years. We agree that collective action is required to ensure students have access to immediate and effective treatment, with routine prevention and intervention as joint strategies. Last but not least, we must value, foster, and commit to shed light on the interaction of food allergy and rhinitis, explore more carefully differences in prediction models of risk factors for LRTI, and validate and improve the model in larger sample sizes and more populations.

## Data Availability Statement

The raw data supporting the conclusions of this article will be made available by the authors, without undue reservation.

## Ethics Statement

The studies involving human participants were reviewed and approved by the Ethics Committee of Beijing University of Chinese Medicine. Written informed consent to participate in this study was provided by the participants' legal guardian/next of kin.

## Author Contributions

ZZ planned and designed the study and directed its implementation. ZZ and WN drafted the protocol. MX, QW, YZ, BP, and MY obtained statutory and ethics approvals. MX and QW contributed to data acquisition. MX and WN conducted statistical analyses and wrote the manuscript. MX, QW, YZ, BP, MY, and XD did the data preparation and quality control. All authors read and approved the final manuscript prior to submission.

## Conflict of Interest

The authors declare that the research was conducted in the absence of any commercial or financial relationships that could be construed as a potential conflict of interest.

## Publisher's Note

All claims expressed in this article are solely those of the authors and do not necessarily represent those of their affiliated organizations, or those of the publisher, the editors and the reviewers. Any product that may be evaluated in this article, or claim that may be made by its manufacturer, is not guaranteed or endorsed by the publisher.
